# The Identification of Intraoperative Risk Factors Can Reduce, but Not Exclude, the Need for Completion Thyroidectomy in Low-Risk Papillary Thyroid Cancer Patients

**DOI:** 10.1089/thy.2019.0274

**Published:** 2020-02-14

**Authors:** Steven J. Craig, Andrew M. Bysice, Steven C. Nakoneshny, Janice L. Pasieka, Shamir P. Chandarana

**Affiliations:** ^1^ Section of General Surgery, Department of Surgery, University of Calgary, Calgary, Canada.; ^2^ Cumming School of Medicine, University of Calgary, Calgary, Canada.; ^3^ Ohlson Research Initiative, Arnie Charbonneau Cancer Institute, Cumming School of Medicine, University of Calgary, Calgary, Canada.; ^4^ Section of Surgical Oncology, Department of Surgery, University of Calgary, Calgary, Canada.; ^5^ Section of Endocrinology, Department of Medicine, University of Calgary, Calgary, Canada.; ^6^ Section of Otolaryngology—Head and Neck Surgery, Department of Surgery, University of Calgary, Calgary, Canada.

**Keywords:** low-risk, risk factors, papillary thyroid cancer, completion thyroidectomy

## Abstract

***Background:*** The extent of initial surgical resection for low-risk papillary thyroid cancer (PTC) remains debatable. Since the 2015 American Thyroid Association (ATA) guidelines, several retrospective studies have reported that 40–60% of patients initially treated with lobectomy would require a completion thyroidectomy (CTx) due to high-risk pathological features (HRFs). These studies are limited by variable preoperative stratification and inability to quantify the value of intraoperative assessment. The study objectives were to determine whether diligent preoperative and intraoperative assessment improves the appropriateness of initial surgery for low-risk PTCs and whether varying the criteria for lobectomy reduces the need for CTx.

***Methods:*** A prospectively collected province-wide database was analyzed over a 10-year period (2008–2017) for patients who underwent a total thyroidectomy (TT) for PTC without preoperative HRFs. All patients had preoperative ultrasound and fine-needle aspirates. Unique to this database are mandatory synoptic operative fields that identify intraoperative risk factors such as positive lymph nodes and local invasion.

***Results:*** In total, 74% of patients (709/959) were deemed eligible for lobectomy. Of those eligible, 149 (21%) had intraoperative risk factors that would necessitate conversion to TT at the initial operation. A further 209 (30%) would require CTx due to HRFs on final pathology. Varying the preoperative criteria for lobectomy did not significantly affect intraoperative conversion or CTx rates.

***Conclusions:*** Although intraoperative assessment reduced the need for CTx in 21%, up to 30% of patients would still require a second operation. Altering the preoperative criteria does not influence this outcome. Patients deemed eligible for lobectomy should be informed that despite careful pre- and intraoperative assessment, there is up to a 30% risk of requiring CTx.

## Introduction

A marked increase in the worldwide incidence of thyroid cancer has in part been attributed to the increased detection of smaller low-risk papillary thyroid cancers (PTCs) (1–3). Despite increased rates of detection, thyroid cancer-related mortality in well-differentiated PTCs has not changed significantly (4). Patients affected by PTCs have a 10-year survival rate exceeding 98% (5). This has led to concern about over-diagnosis and over-treatment of otherwise low-risk PTCs and to advocacy of less aggressive treatment paradigms (6–8).

In tradition, total thyroidectomy (TT) was considered the standard of care for patients with PTCs >1 cm (9,10). However, in 2015 the American Thyroid Association (ATA) changed its guidelines for the initial operative approach to low-risk PTCs, defined as 1–4 cm tumors without high-risk pathological features (HRFs) (Table 1), to allow for either lobectomy or TT (11). This change was based on several retrospective studies that demonstrated equivalent survival outcomes in patients undergoing a lobectomy versus TT (12–15). Coinciding with the move toward less aggressive initial surgery for low-risk PTCs, there has also been an evidence-based trend toward more selective use of radioactive iodine (RAI) in low and intermediate risk patients (11,16,17).

**Table 1.** Identifiable High-Risk Features That Would be Considered an Indication for Either Intraoperative Conversion to Total Thyroidectomy, or Completion Thyroidectomy

Intraoperative features for conversion to TT	Postoperative features for CTx
ETE	ETE
Positive lymph nodes	VI
	Positive lymph nodes
	Positive tumor margins
	Index tumor >4 cm
	Aggressive histopathology

CTx, completion thyroidectomy; ETE, extrathyroidal extension; TT, total thyroidectomy; VI, vascular invasion.

Since the publication of the 2015 ATA guidelines, several retrospective studies have estimated the theoretical completion thyroidectomy (CTx) rate in low-risk PTC patients if initially treated with lobectomy, based on the identification of HRFs in TT specimens. These studies have reported that between 40% and 60% of patients with low-risk PTCs would require a CTx due to HRFs (18–23). However, these studies are retrospective and may be limited by incomplete reporting of preoperative ultrasound (US) features and patient history. Furthermore, these studies were unable to directly quantify the value of the surgeon's intraoperative assessment for HRFs that would necessitate conversion from lobectomy to TT at the initial operation, potentially sparing a second operation.

The 2015 ATA recommendations have, therefore, posed two challenging questions for clinicians. First, in patients with seemingly low-risk PTCs initially managed with lobectomy, what is the likelihood of occult HRFs, recognized either intraoperatively or postoperatively, which would necessitate the removal of the entire thyroid? Second, which preoperative factors should clinicians use to determine patient suitability for lobectomy? To answer these questions, we used a province-wide prospectively collected surgical database incorporating a unique synoptic operative reporting system. Our primary objectives were (I) to determine the prevalence of intraoperative HRFs and their impact on reducing the need for CTx and (II) to determine whether varying the preoperative selection criteria for lobectomy could reduce the need for CTx.

## Methods

### Patients

A prospectively collected province-wide database from Alberta, Canada, was analyzed for a 10-year period (2008–2017) for patients who underwent a TT for PTCs. Data were provided by 20 surgeons from across the province of Alberta, including academic and community practices, and low- and high-volume surgeons. Unique to this surgical comprehensive operative database are mandatory operative fields that prospectively identify intraoperative risk factors such as worrisome metastatic lymph nodes (LNs; i.e., not detected preoperatively) and local invasion, suggesting extrathyroidal extension (ETE). The data points in the synoptic database system are all entered by the attending surgeon at the time of operation, and mandatory fields require completion to finish the operative report. This synoptic operative report was developed to ensure that a negative finding could not be implied through this utilization of mandatory fields requiring either a positive or negative result (24). A second province-wide clinical database allowed for analysis of all preoperative US results, preoperative fine needle aspirate (FNA) cytology, and postoperative histopathology. Every data point included in this study was cross-checked by a practicing surgeon for accuracy, validity, and quality control.

The study inclusion criteria were adult patients (aged >18 years at date of surgery) who underwent a thyroidectomy with a confirmed Bethesda category 5 or 6 PTC lesion on preoperative FNA during the study period (2008–2017). We excluded any patient who did not have a preoperative US result, those with papillary microcarcinoma, and those patients who had preoperative clinically node positive disease or distant metastatic disease.

Assessment for eligibility for lobectomy was based on the 2015 ATA guidelines: preoperative US findings (1–4 cm in size without ETE or suspicious LNs) and the absence of family history or radiation exposure. Unique to our synoptic database is that family and radiation history are mandatory fields; therefore, patients with such history were able to be confidently excluded from our cohort. US reports were reviewed by the study authors for lesion size and to confirm the absence of contralateral atypical nodules, suspicious LNs, or local invasion. Given the study period, the operation of choice for a low-risk PTC 1–4 cm during part of the study period was TT +/− prophylactic central lymph node dissection (pCLND), and as such pCLND results were included in the data collection.

### Indications for intraoperative conversion to TT

This prospective synoptic operative database included mandatory fields that captured data on intraoperative assessment for (I) evidence or suspicion of invasion into local structures (including muscle, recurrent laryngeal nerve, esophagus, trachea, or other structures) and (II) metastatic LNs confirmed on intraoperative frozen section analysis. Invasion into local structures was based on the operating surgeon's assessment of gross adherence to adjacent structures. In the cohort of patients who were initially eligible for lobectomy, if either of these criteria were positive, these patients were deemed to have theoretically required an intraoperative conversion to a TT.

### Indications for CTx

Final histopathology reports were reviewed to identify any HRFs (Table 1) that would by ATA criteria be deemed to be intermediate to high risk of recurrence. If any one of these HRFs were identified on final histopathology, the patient was deemed to have required a CTx to facilitate RAI therapy. ETE included both microscopic and macroscopic extensions. LNs were considered positive with either micrometastases or macrometastases.

### Analysis

Statistical analysis was performed using Stata, version 15.1 (Stata Corp., College Station, Texas). Descriptive statistics were used to report rates of intraoperative conversion to TT and of CTx. To determine the impact of preoperative parameters on rate of CTx, subset analyses were also performed on groups presumed to be at lower risk of having HRFs; these groups were based on altering the preoperative selection criteria for lobectomy, such as including patients with smaller tumor size and younger patient age. The study was approved by the local health research ethics board of the Alberta-Cancer Committee (Ethics no. 25992).

## Results

**FIG. 1.**
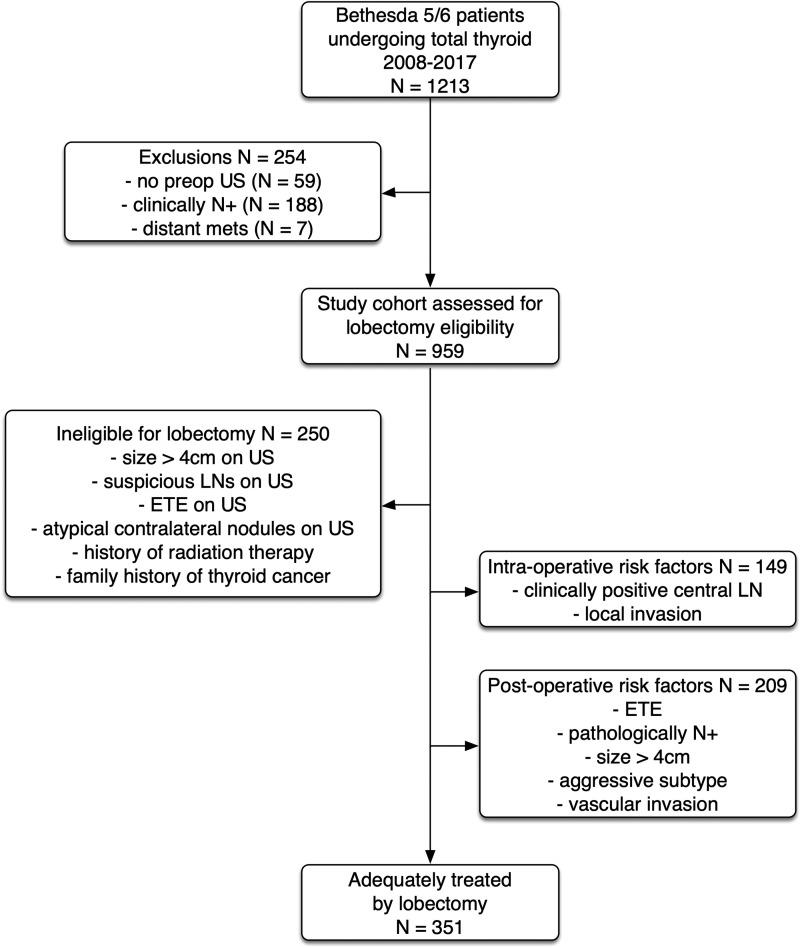
Flowchart of patient exclusions and eligibilities. ETE, extrathyroidal extension; LN, lymph node; US, ultrasound.

We identified 1,213 adult patients (aged >18 years at date of surgery) who underwent a TT with a confirmed Bethesda category 5 or 6 PTC lesion on preoperative FNA during the study period. We excluded 254 patients (21%) because they could not be assessed for, or would not have been eligible for, an initial lobectomy; either they did not have a preoperative US result available (59 patients) or preoperatively they had clinically positive nodal disease (188 patients) or distant metastases (7 patients). This resulted in 959 patients who met our eligibility criteria (Fig. 1). Seventy-four percent (709/959) were found to be eligible for initial lobectomy, based on application of the ATA 2015 guidelines. Reasons for ineligibility for lobectomy in the 250 patients were size >4 cm (117 patients) on preoperative US, suspicious central neck LNs on preoperative US despite no clinically apparent LN disease (57 patients), evidence of ETE on preoperative imaging (10 patients), history of preoperative radiation therapy (35 patients), and family history of thyroid cancer (51 patients). Twenty patients had more than one reason for ineligibility for lobectomy. A total of 709 patients who were deemed eligible for initial lobectomy had a mean patient age of 46.5 years (range 18–89 years) and 81% of patients were female (575/709). The average tumor size on preoperative US was 2.09 cm (standard deviation [SD] ±0.83 cm).

Of the 709 patients, 149 (21%) had an intraoperative finding that would necessitate conversion to TT during the initial operation. For 104 patients, the reason for intraoperative conversion was evidence of local invasion, and for 45 patients it was the discovery of worrisome LNs confirmed as metastatic on frozen section. Correlation with final pathology was 57% for ETE and 87% for LN positivity. Thus, of the 709 patients initially eligible for lobectomy based on preoperative criteria, 560 patients would have ultimately completed initial lobectomy after intraoperative assessment.

**FIG. 2.**
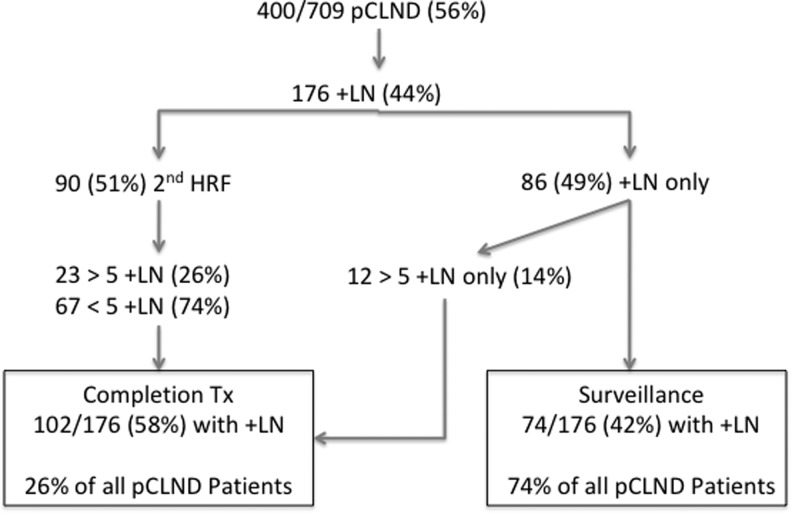
Subanalysis of patients undergoing pCLND. HRF, high-risk pathological feature; pCLND, prophylactic central lymph node dissection.

Postoperatively, a further 209 patients (30%) would be considered for a CTx due to presence of one or more HRFs on final histopathology. Thus, of the 709 patients initially eligible for lobectomy, only 351 (49%) patients would have adequately been treated with lobectomy (Fig. 1). The high-risk findings on histopathology necessitating CTx were ETE (64 patients), positive LNs (148 patients), tumor size >4 cm (13 patients), aggressive histological subtype (15 patients), and vascular invasion (56 patients). The cumulative frequency of these risk factors is shown in Table 2. There were no differences across both sexes with respect to frequency of HRFs. A subanalysis of patients who underwent pCLND (level VI) demonstrated that 35/400 (8%) of pCLNDs had >5 positive LNs. An additional HRF (beyond LN metastases) was found in 23 of these patients, resulting in 12 (3%) of patients for whom the pCLND yielding >5 LN was the only HRF (Fig. 2).

**Table 2.** Distribution of High-Risk Factors on Histopathology in 209 Patients Who Would Have Required Completion Thyroidectomy

Risk factors	n (%)	Risk factor summary	Count
1	144 (68.9)	pN+	93
		ETE	25
		>4 cm	15
		VI	8
		Histo	3
2	47 (22.5)	VI & pN+	19
		ETE & pN+	17
		VI & ETE	5
		VI & Histo	2
		pN+ & Histo	2
		ETE & Histo	2
3	14 (6.7)	VI & ETE & pN+	8
		VI & pN+ & >4 cm	3
		ETE & pN+ & Histo	2
		ETE & >4 cm & Histo	1
4	4 (1.9)	VI & ETE & pN+ & Histo	3
		VI & ETE & pN+ & >4 cm	1

>4 cm, size greater than 4 cm; histo, aggressive histological subtype; pN+, lymph node positive disease on pathology; VI, vascular invasion.

The average tumor size on final histology was 1.87 cm (range 0.1–6.5 cm, SD 1.01 cm). This was smaller than the average tumor size (2.09 cm) on preoperative US. There were 90 patients who had a lesion measuring >1 cm on US, which on final histopathology measured <1 cm (papillary microcarcinoma). Of the 90 patients with a tumor size <1 cm of final histopathology, 21 (23%) also had 1 or more HRFs. There were also 8 patients who had a benign lesion on final histopathology, despite having a Bethesda category 5 or 6 lesion on FNA.

Restricting the preoperative criteria (by age and tumor size) for lobectomy resulted in a significant reduction in the number of patients felt to be initially eligible for lobectomy; only 25–39% of the cohort were eligible (Table 3). However, adjusting the preoperative criteria did not have a statistically significant effect on intraoperative conversion or CTx rates when compared with the ATA guideline criteria. Limiting age to ≤55 years and reducing nodule size to 1–3 cm resulted in a conversion rate of 20% and CTx rate of 33% (*p* = 0.59). Further reducing nodule size to 1–2 cm, and varying the age, resulted in conversion and completion rates of 16% and 29% for age ≤45 years (*p* = 0.15), and 18% and 28% for age ≤55 years (*p* = 0.16), respectively (Table 3).

**Table 3.** The Effect of Varying Preoperative Criteria for Lobectomy on Intraoperative Conversion Rate and Completion Thyroidectomy Rate

Criteria	No. eligible	Conversion rate	Completion rate	Combined rate	p
ATA (*n* = 709) (any age, size 1–4 cm)	74%	21% (149/709)	30% (209/709)	51% (358/709)	Reference for comparison
Criteria 2 (*n* = 235) (age <50, size 1–3 cm)	25%	20% (47/235)	33% (77/235)	53% (124/235)	0.55
Criteria 3 (*n* = 276) (age <55, size 1–2 cm)	29%	18% (51/276)	28% (78/276)	46% (129/276)	0.18
Criteria 4 (*n* = 174) (age <45, size 1–2 cm)	18%	16% (28/174)	29% (51/174)	45% (79/174)	0.26

ATA, American Thyroid Association.

## Discussion

Our analysis of a prospective province-wide database shows that despite strict preoperative selection, 51% of patients with a low-risk 1–4 cm PTC initially meeting criteria for lobectomy would ultimately require their entire thyroid to be removed. Although in reality identification of intraoperative risk factors obviated the need for CTx in 21% of patients, our series still found 30% of patients would be deemed ATA intermediate or high risk, requiring a second operation for a CTx. This underscores the importance of meticulous intraoperative assessment by the surgeon performing a lobectomy for PTCs. Given that our database was derived from >20 surgeons across diverse practice settings and comprised 709 patients with confirmed preoperative eligibility for lobectomy, these figures are likely to accurately reflect the risk of intraoperative conversion and postoperative CTx in clinical practice. As such, they are important empirically derived estimates that can be used to inform consent processes for patients considering initial lobectomy.

Our results are consistent with previous retrospective studies that have estimated that 40–60% of patients with low-risk 1–4 cm PTCs, if initially treated with lobectomy, would require a CTx due to HRF in postoperative pathology reports (18–23). However, a strength of this study is that it provides a uniform and realistic study population, limiting the study cohort to only those patients who had (I) adequate preoperative FNA tumor cytology; (II) documented preoperative US size, features, and LN assessment; and (III) documented absence of a family history of thyroid cancer and radiation exposure. Kluijfhout *et al.* determined tumor size from final histopathology for study inclusion rather than preoperative US (21). In other studies it is unclear whether the reported tumor size was determined by preoperative US or final histopathology (20), whether radiation exposure and family history were accounted for in their preoperative risk stratification (19,22), or whether the patients studied were confirmed Bethesda 5 or 6 lesions on preoperative FNA (20,21).

Given the high rates of CTx cited in this study and in the literature, identifying preoperative factors that predict HRFs on postoperative histopathology could allow for better patient selection for initial lobectomy, and ultimately reduce the CTx rate. Although previous studies have shown that tumor size and age are preoperative factors associated with a lower risk of requiring CTx (19,21), others have reported that the prognostic influence of tumor size in PTCs only applies to those aged >55 years (25). Although altering the variables did reduce the percentage of the cohort eligible for lobectomy, we found that varying the preoperative tumor size and age criteria for lobectomy did not have a significant effect on either the intraoperative conversion rate or CTx rate (Table 3). This illustrates that the HRFs that determine the need for CTx are poorly predicted by preoperative clinical parameters.

The indication for CTx in well-differentiated thyroid cancer is to facilitate the administration of postoperative RAI. In recent years, there has been an evidence-based trend toward selective use of RAI (26). The evidence for determining what constitutes a HRF has become “dynamic” and often depends on local practices and guidelines that may lead to varying levels of CTx over time. For example, in the 2009 ATA guidelines, minimal ETE or vascular invasion were considered “intermediate” risk features, for which RAI would be recommended. Whereas in the 2015 ATA guidelines, these same features were changed to “low” risk features based on contemporary evidence of the impact of ETE, altering the recommendations for RAI (9,11,16). There are also discrepancies in interpretation of HRFs between regions; the 2014 British Thyroid Association guidelines recognize positive margin and multifocality as HRFs, whereas the 2015 ATA guidelines do not (11,17). As such, surgeons need to be aware of local treatment protocols and how the interpretation of HRFs may influence the recommendation for RAI, necessitating the need for CTx rate in presumed low-risk patients treated with initial lobectomy.

To align our study with previous retrospective studies that have evaluated CTx rate, positive LN status was included in our primary analysis as an HRF requiring CTx. This was also consistent with the ATA 2009 guidelines, which governed much of the study period, and would recommend RAI for patients with positive LNs. A total of 187 patients had positive LNs. Thirty-nine of these patients were converted intraoperatively to a TT because of intraoperative findings, including identification of positive LNs. This resulted in 148 postoperative “lobectomy” patients wherein positive LNs were demonstrated on final pathology. In 93 of these patients, a positive LN was their only HRF. This likely overestimated the CTx rate in our study as LN positivity is also a dynamic risk factor. pCLND was part of the standard of care during the early parts of the study period (2009–2013), and although pCLND is no longer routinely recommended, central compartment LNs may still be found with a lobectomy or TT specimen. The 2015 ATA guidelines differ from the 2009 guidelines in that they do not simply consider whether or not there are positive nodes, but assign a risk based on the number (>5) and size of positive LNs, as well as the presence of extranodal extension. The combination of having a period of time when pCLND was standard practice and the lack of detail on LN pathology made it difficult to stratify the LN risk status beyond simply “positive” or “negative.” In the overall calculation, if any “positive LN” was removed from the list of HRFs that trigger CTx, the theoretical CTx rate would decrease from 31% to 21% (Table 2).

It has been well documented that pCLND not only increases the number of microscopically positive LNs detected, but also allows for a robust number of nodes recovered to utilize the current guidelines of >5 positive nodes as an intermediate risk criteria (11,27,28). In a subanalysis of the 400 patients in our cohort who underwent a pCLND, we found that 176 had documented positive LNs. For half of these pCLND patients (*n* = 86), LN positivity was the only risk factor found, and the other 51% had an additional HRF on final pathology (Fig. 2). DiMarco *et al.* found that of their 275 patients eligible for lobectomy who had undergone a TT and pCLND, 5% had >5 positive LNs as their only risk factor for CTx on final pathology (23). This is in keeping with our pCLND data that found 12 (3%) of all pCLND patients had >5 positive LNs as their only risk factor (Fig. 2). Even with pCLND providing a more robust LN assessment and a better stratification of HRFs, 26% of these patients would have required CTx.

This study has several limitations. First, this a retrospective analysis of a prospectively collected database, which carries an inherent potential selection bias and may fail to accurately capture all data pertinent to the treatment decisions of surgeons and patients. Second, the reporting of pathology changed during the study period as comprehensive synoptic pathology reports were adopted. The pathological details about the size of LN metastatic deposits or presence of extranodal extension were often absent in our reports because routine detailed pathological synoptic reporting had only in recent years been fully implemented at our institution. This limited our ability to analyze the effect of subtle pathology characteristics including the degree of ETE and LN features. This likely has led to an overestimation of the number of patients requiring CTx. Third, the definition and interpretation of HRFs are dynamic. Some of the HRFs defined in our study design may have unclear clinical significance as newer evidence becomes available, particularly regarding LN pathology and ETE (16,28). Fourth, while our analysis allows for calculation of theoretical rates of conversion and CTx, it does not include data on outcomes for these patients, such as rates of injury to the recurrent laryngeal nerve and parathyroid glands. Finally, this analysis does not take into account patient preference or other issues at the time of consent that may have resulted in a TT at initial assessment. However, the strength of this study lies in the uniqueness of our synoptic operative database. The data from this database is entered by the operating surgeon shortly upon completion of the operation. The required mandatory information regarding preoperative radiation and family history, as well as the intraoperative assessment and concerns of local invasion, provides a more realistic assessment of the surgical intraoperative findings that cannot be captured in a retrospective cancer registry database or by reading a dictated operative report.

In summary, we identified that up to 50% of patients deemed preoperatively to be low-risk PTCs for whom a lobectomy alone would be adequate may ultimately require a TT. Surgical conversion at the time of the operation was found in 21% of low-risk patients. Depending on the stratification of intermediate and HRFs, between 21% and 30% additional patients would require a CTx.

Careful preoperative risk stratification is required to identify patients with low-risk PTCs who would be adequately treated by an initial thyroid lobectomy. However, low-risk PTC patients deemed eligible for lobectomy should be informed at the time of consent that despite diligent preoperative and intraoperative assessment, there is a risk of requiring a CTx that may be as high as 30%. In addition, surgeons, endocrinologists, and patients should be aware that in ∼20% of patients deemed eligible for lobectomy preoperatively, an intraoperative HRF will be identified, resulting in a conversion to TT if deemed safe.
